# Association of cigarette smoking with neuromyelitis optica-immunoglobulin G sero-positivity in neuromyelitis optica spectrum disorder

**Published:** 2019-07-06

**Authors:** Sharareh Eskandarieh, Abdorreza Naser Moghadasi, Mohammad Ali Sahraiain, Amir Reza Azimi, Negar Molazadeh

**Affiliations:** Multiple Sclerosis Research Center, Neuroscience Institute, Tehran University of Medical Sciences, Tehran, Iran

**Keywords:** Neuromyelitis Optica, Cigarette, Neuromyelitis Optica-Immunoglobulin G

## Abstract

**Background:** Neuromyelitis optica spectrum disorder (NMOSD) is a neuroinflammatory demyelinating disease caused by the presence of a highly specific serum autoantibody marker, NMO-immunoglobulin G (NMO-IgG), that reacts against the water channel aquaporin-4 (AQP4). The present study examined the association between NMO-IgG sero-positivity and environmental factors such as cigarette smoking.

**Methods:** A cross-sectional study was conducted in Sina Hospital, a tertiary referral center in Tehran, Iran. All the patients with a definite diagnosis of NMOSD were involved in this study. The enzyme-linked immunosorbent assay (ELISA) was used to examine the AQP4-IgG status. To assess the association between NMO-IgG sero-positivity and cigarette smoking, a researcher-made questionnaire covering patients’ lifestyle information on smoking habits was designed and administered using the structured face-to-face interviews with the patients.

**Results:** The positive and negative NMO-IgG results were found in 44 (46.8%) and 50 (53.2%) patients, respectively. The increased NMO-IgG sero-positivity odds were observed among the lifetime smokers [odds ratio (OR) = 3.24, 95% confidence interval (CI): 1.16-9.08], current smokers (OR = 6.08, 95% CI: 1.26-29.39), and passive smokers (OR = 2.22, 95% CI: 1.10-4.50).

**Conclusion:** Lifetime and current smoking as well as passive smoking can be regarded as risk factors for NMO-IgG sero-positivity. Smoking with its immunological effects can lead to the production of autoantibodies such as NMO-IgG.

## Introduction

Neuromyelitis optica spectrum disorder (NMOSD) is an infrequent autoimmune inflammatory disorder of the central nervous system (CNS).^1^ The prevalence of NMOSD among the Iranian population living in Tehran, Iran, was 0.86 per 100000 populations in 2016. Moreover, the highest prevalence rate was observed in the 40-49 years age group of the mentioned population.^2^ Despite the remarkable number of studies addressing NMOSD, its risk factors have not been thoroughly attended.^3^ Smoking has been recognized as a major environmental risk factor promoting the development of a wide range of inflammatory and autoimmune diseases in genetically-susceptible individuals.^4^ The mechanism by which cigarette smoking causes an imbalance in the immune system and contributes to the pathogenesis of autoimmunity has not been grasped yet; however, some factors such as toxic compounds in cigarettes that cause epigenetic changes seem to be involved in this regard.^5^^,^^6^

Rheumatoid arthritis (RA),^7^ systemic lupus erythematosus (SLE),^8^ multiple sclerosis (MS),^9^ NMOSD,^3^ and Graves’ hyperthyroidism^10^ are among the autoimmune diseases that smoking has been demonstrated to play a role in their pathogenesis. Interestingly, smoking also leads to autoantibody production, which can be regarded as a piece of its immune balance impairment puzzle. NMOSD is distinguished from MS with the recognition of aquaporin-4 immunoglobulin G (AQP4-IgG), also known as NMO-IgG, which acts as an autoantibody against AQP4 that is a water channel in the brain parenchyma.^11^ As far as the authors of the present study are concerned, the association between smoking and NMO-IgG sero-positivity has not been investigated yet. Therefore, the present study aimed at determining the smoking history of patients with NMOSD and then examining any association between smoking and NMO-IgG sero-positivity.

## Materials and Methods

***Study population and patients:*** A cross-sectional study was conducted in Sina Hospital, a tertiary referral center in Tehran.^2^^,^^12^ All the patients, who received a definite diagnosis of NMOSD based on the international 2015 consensus criteria^1^^,^^13^ and were admitted to the hospital from August 1, 2015 to September 21, 2016, were included in the study. The diagnosis was confirmed by a neurologist, as well. The NMOSD patients with positive history of smoking were compared with a group of NMOSD patients without any smoking habits.

***Laboratory analyses/detection of NMO-IgG:*** The enzyme-linked immunosorbent assay (ELISA) was used to examine the presence of AQP4-IgG status.^14^ Most of the tests were performed during the relapse phase, before receiving corticosteroids.

***Cigarette smoking:*** To assess the possible association between NMO-IgG sero-positivity and cigarette smoking habits, a researcher-made questionnaire was designed, which covered the patients’ demographic information, lifestyle information on smoking habits, and clinical characteristics including the onset of the clinical symptoms and the status of NMO-IgG. To collect the required data, structured face-to-face interviews were conducted by trained interviewers. Data about patients’ lifetime cigarette smoking status, number of cigarettes per day, and years of smoking were collected using the following three categories: lifetime daily cigarette smoking (1 per day for over 6 months), current daily cigarette smoking, and passive smoking (whether the person lived with a regularly smoking person or has been with a regular smoker in the workplace).^3^

Chi-square test was used to examine the association among the variables, and logistic regression was applied to determine the interactions between the variables. The crude odds ratio (OR), adjusted OR (AOR), and 95% confidence interval (CI) were calculated. P-values < 0.05 were considered significant. All data analyses were performed using SPSS software (version 23, IBM Corporation, Armonk, NY, USA). 

The study protocol was approved by the Ethics Committee of Institutional Review Board (IRB) in Tehran University of Medical Sciences, Tehran (reference number: IRTUMSREC13941195). Moreover, informed consents were obtained from all patients.

## Results

A total of 94 patients consisting of 78 (83%) women and 16 (17%) men were enrolled in the study. Female to male ratio was 4.8:1 (Table 1). The mean patient age on the prevalence day was 37.05 years. The minimum and maximum ages were 14 and 71 years, respectively. The patients aging 19-38 years comprised the major proportion of the participants (52.1%) (Table 1).

**Table 1:** Baseline characteristics and clinical features of patients with neuromyelitis optica spectrum disorder (NMOSD)

**Variables**	**Women [n (%)]**	**Men [n (%)]**	**Total [n (%)]**	**P**
Age group (year)				0.26
≤ 18	2 (2.6)	2 (12.5)	4 (4.3)	
19-38	42 (53.8)	7 (43.7)	49 (52.1)	
39-58	32 (41.0)	6 (37.5)	38 (40.4)	
≥ 59	2 (2.6)	1 (6.3)	3 (3.2)	
Marital status				0.57
Single	27 (34.6)	7 (43.8)	34 (36.2)	
Married	51 (65.4)	9 (56.3)	60 (63.8)	
Educational level				0.29
Illiterate	3 (3.8)	2 (12.5)	5 (5.3)	
Primary/secondary school	35 (44.9)	8 (50.0)	43 (45.7)	
University	40 (51.3)	6 (37.5)	46 (48.9)	
Clinical onset symptom				0.14
ON	25 (32.1)	4 (25.0)	29 (30.8)	
TM	20 (25.6)	8 (50.0)	28 (29.8)	
ON + TM	33 (42.3)	4 (25.0)	37 (39.4)	
NMO-IgG status				0.29
Positive	38 (48.7)	6 (37.5)	44 (46.8)	
Negative	40 (51.3)	10 (62.5)	50 (53.2)	

ON: Optic neuritis; TM: Transverse myelitis; NMO-IgG: Neuromyelitis optica-immunoglobulin G

Moreover, the mean age of the patients with NMOSD at the disease onset was 31.88 years with a mode of 42 years. There was no significant relationship between the patients’ mean age at disease onset and their gender (P = 0.57). Majority of the patients, 60 (63.8%), were married, and 46 (48.9%) ones had a university degree.

The status of NMO-IgG was examined in all patients. The results were negative and positive for 50 (53.2%) and 44 (46.8%) patients, respectively. The first presenting symptoms among patients were symptoms of transverse myelitis (TM), optic neuritis (ON), and TM + ON that were manifested in 28 (29.8%), 29 (30.8%), and 37 (39.4%) patients, respectively (Table 1).

The results of the univariate analysis addressing the NMO-IgG status as well as the significant association of environmental risk factors among NMOSD population are presented in table 2. There were increased odds of NMO-IgG sero-positivity status among patients with lifetime cigarette smoking habit for at least six months (AOR = 4.12, 95% CI: 1.01-16.86) (Table 2). Following the mentioned group, the patients with lifetime passive smoking status indicated the increased odds of NMO-IgG sero-positivity status (AOR = 3.06, 95% CI: 1.31-7.13) (Table 2).

**Table 2:** Environmental risk factors for neuromyelitis optica-immunoglobulin G (NMO-IgG)

**Variables**	**NMO-IgG**	**NMO-IgG**	**Crude OR (95% CI)**	**AOR (95% CI)**	**P**
	**Positive [n (%)]**	**Negative [n (%)]**			
Age group (year)					0.53
≤ 18	2 (4.5)	6 (12.0)	Reference	Reference	
19-38	29 (65.9)	31 (62.0)	0.35 (0.06-1.90)	0.37 (0.06-1.99)	
39-58	12 (27.3)	12 (24.0)	0.33 (0.05-1.99)	0.39 (0.06-2.39)	
≥ 59	1 (2.3)	1 (2.0)	0.33 (0.01-8.18)	0.30 (0.01-7.61)	
Lifetime cigarette smoking					0.03
Yes	3 (6.8)	12 (24.0)	4.31 (1.13-16.48)	4.12 (1.01-16.86)	
No	41 (93.2)	38 (76.0)	Reference	Reference	
Lifetime passive smoking					0.01
Yes	18 (40.9)	33 (66.0)	2.80 (1.21-6.48)	3.06 (1.31-7.13)	
No	26 (59.1)	17 (34.0)	Reference	Reference	

Adjusted for sex

OR: Odds ratio; AOR: Adjusted odds ratio; CI: Confidence interval; NMO-IgG: Neuromyelitis optica-immunoglobulin G

## Discussion

The present study revealed a direct association between long-term smoking and antibody positivity in patients with NMOSD. Although the relationship between antibody titer and disease severity still remains imprecise and the provided results are contradictory in this respect,^15^^,^^16^ considering the role of antibodies in the pathogenesis of the disease as well as the evidence presented in the pertinent studies, the relationship between antibodies and severity of the disease is indicated.^17^

The obtained finding is of great value as it can confirm the findings of previous studies specifying the relationship between smoking and disease onset. In the study conducted by Heydarpour et al., it was indicated that current use of cigarette directly correlated with the number of attacks. Furthermore, duration and daily dose-rate of cigarette smoking were directly related to the patients’ Expanded Disability Status Scale (EDSS) score.^18^ Moreover, it has been revealed that smoking has a direct relationship with the mentioned patients’ quality of life.^19^ To the researchers’ best knowledge, the relationship between cigarette smoking and antibodies has not been investigated yet. However, reviewing similar studies focusing on a wide array of autoimmune diseases can shed more light on the cause of the observed relationship. As it was mentioned in the introduction, cigarette smoking has specific effects on the immune system and generally can lead to immunosuppression.^20^

It seems that the mentioned effect of smoking is mainly due to its nicotine, which has inhibitory effects on both innate and adaptive immune responses.^21^ Smoking also has diverse effects on the components of the immune system. Smoking increases the level of T-helper 17 (Th17) cells in the blood of patients with chronic obstructive pulmonary disease (COPD)^22^ and Crohn's disease,^23^ and subsequently worsens the disease-specific health status. Moreover, smoking can increase the number and activity level of cytotoxic T cells (CD8+ T cells) in the blood.^24^

Smoking not only affects T cells but also has a definite effect on B cells. In this regard, smoking can lead to an increased number of memory B cells, which is followed by an increase in the level of IgE and a decrease in the number of regulatory B cells (Bregs).^25^ As already mentioned, smoking has a definite effect on the innate immunity as a result of increasing the incidence rate and impact intensity of toll-like receptors (TLRs)^26^ as well as affecting the performance and efficiency of dendritic cells (DCs), natural killer cells (NK cells), and macrophages.^25^ The mentioned inclusive effect of smoking on the immune system practically anticipates its relationship with antibody positivity in patients. As already specified, to the best of the authors’ knowledge, no research has been conducted in this regard so far. However, the effect of smoking on the production of antibodies in a number of other autoimmune diseases has been previously examined, and the results revealed that current use of cigarettes was directly related to double stranded deoxyribonucleic acid (dsDNA) autoantibody sero-positivity. According to the theory presented by the researchers interested in this area of investigation, DNA damage caused by smoking can lead to an increased risk of antibody production against smoking, which can be considered as a justification for the obtained findings. 

The role of smoking in the production of antibodies can be probably facilitated by antigen expression. The mentioned issue has not been only attended to by the authors of the present study, but also is insisted on in other studies. For instance, the study conducted by Mysikova et al. revealed that the level of antibodies specific for New York esophageal squamous cell carcinoma 1 (NY-ESO-1) in patients with non-small-cell lung cancer (NSCLC) was significantly higher than that of the control group. The mentioned finding indicates that the level of antibodies has a direct relationship with smoking. Probably, the immunological effects of smoking lead to antigen presentation and subsequently antibody production.^27^ The presented studies can be used as a guide to explain the findings of the present study regarding the relationship between sero-positivity and smoking in patients with NMOSD.

Conceivably, smoking with its immunological effects leads to other additional presentations of antibodies. AQP4 is a membrane protein that is predominately expressed in the end-feet of astrocytes.^28^ The mentioned point clarifies why astrocytes play a very significant role in NMOSD, which is referred to as astrocytopathy.^29^ It has been indicated that smoking can damage the mentioned cells^30^ which probably contribute to the expression of AQP4 and consequently development of antibodies. Moreover, animal studies conducted in this regard verify the mentioned claim. It has been reported that following the onset of smoke inhalation in rats, the level of AQP4 expression in astrocyte cells in the retina increased.^31^ Considering the findings of the present study as well as those of the mentioned studies, it can be concluded that smoking has the same effect on human brain astrocytes. 

The present study can be considered as an introduction to the effect of smoking on the trend and severity of NMOSD. However, further research is required to present a better and more comprehensive understanding of this issue. Clinically, the presented findings can be regarded as very strong evidence for recommending smoking cessation in patients with NMOSD which can in turn lead to the improvement of disease condition.

## Conclusion

Smoking cigarette leads to the production of autoantibodies such as NMO-IgG in patients with NMOSD. Lifetime and current cigarette smoking habits are the risk factors for NMO-IgG sero-positivity.

## Notes:

**How to cite this article:** Eskandarieh Sh, Naser Moghadasi A, Sahraiain MA, Azimi AR, Molazadeh N. Association of cigarette smoking with neuromyelitis optica-immunoglobulin G sero-positivity in neuromyelitis optica spectrum disorder. Iran J Neurol 2019; 18(3): 93-8.
